# Early risk factors for being a bully, victim, or bully/victim in late elementary and early secondary education. The longitudinal TRAILS study

**DOI:** 10.1186/1471-2458-11-440

**Published:** 2011-06-06

**Authors:** Danielle EMC Jansen, René Veenstra, Johan Ormel, Frank C Verhulst, Sijmen A Reijneveld

**Affiliations:** 1 Department of Health Sciences, University Medical Center Groningen, University of Groningen, PO Box 196, 9700 AD Groningen, The Netherlands; 2 Department of Sociology and Interuniversity Center for Social Science Theory and Methodology (ICS), University of Groningen, Grote Rozenstraat 31, 9712 TG Groningen, The Netherlands; 3 Department of Psychiatry, University Medical Center Groningen, University of Groningen, PO Box 30001, 9700 RB Groningen, The Netherlands; 4 Department of Child and Adolescent Psychiatry, Erasmus Medical Center, PO Box 2060, 3000 CB Rotterdam, The Netherlands; 5 Department of Health Sciences, University Medical Center Groningen, University of Groningen, PO Box 196, 9700 AD Groningen, The Netherlands

**Keywords:** bullying, victimization, risk factors

## Abstract

**Background:**

Data regarding the impact of early risk factors on later involvement in bullying are scarce. We investigated the impact of preschool behaviors, family characteristics (socio-economic status, family breakup) and parental mental health on bullying and victimization at age 11 (T1) and age 13.5 (T2).

**Methods:**

longitudinal data from a subsample of the TRacking Adolescents' Individual Lives Survey (TRAILS) (T1: N = 982; T2: N = 977). TRAILS is a prospective study of adolescent mental health in a mixed urban and rural region of the Netherlands. At T1 parents reported on family characteristics, parental mental health and retrospectively on children's preschool behavior at age 4-5. Schoolmates reported involvement of adolescents in bullying or victimization at T1 and T2.

**Results:**

Children with preschool anxiety were less likely to be bully/victim at T1. Children with preschool aggressiveness were more likely to be bully (T1), bully/victim (T1 and T2) and victim (T2) and children with good preschool motor functioning were more likely to be bully (T1) and less likely to be victim (T1 and T2). Children from low socioeconomic status families were more likely be to be bully, victim, or bully/victim and less likely to be uninvolved both at T1 and T2. Finally, children from intact two parent families were more likely to be uninvolved at T2.

**Conclusion:**

Preschool behavioral, emotional and motor problems, socioeconomic status, and family breakup are related to involvement in bullying at a later age. Prevention of bullying and its consequences can be enhanced by focusing on risk groups in early life.

## Background

Bullying is a serious problem in schools all over the world. Studies show that 20% to 54% of school children are repeatedly involved in bullying as perpetrators and/or as victims [1,2].

Research on bullying mostly focuses on characteristics of children at the moment they already are involved in bullying. Results show that bullies usually have high levels of aggressive and impulsive behavior towards peers [1,3,4]. Victims of bullying tend to be physically weaker, more withdrawn, depressed, anxious, and also less prosocial than uninvolved children [3,5]. Finally, bully/victims, children who bully others and are themselves also victimized by their peers, demonstrate high levels of both aggression and depression, and they score low on measures of academic competence, prosocial behavior, and self esteem [3,4].

Evidence is very limited on the association of characteristics of the children and their environment *before *they were involved in bullying. The limited evidence shows that early emotional and behavioral problems are associated with both bullying and victimization at an advanced age. Schwartz et al [6]found in a 4-year prospective study that early behavior problems predicted later victimization. Also the association between early aggression and later victimization has been documented before [7]. Sourander et al [8]found that having early emotional problems was associated with both bullying and victimization 8 years later. Next to this, Bowes et al [9] showed that early socioenvironmental factors such as domestic violence and problems with neighbors are associated with children' risk for becoming involved in bullying.

Evidence on the association of other early possible risk factors like motor functioning and parental mental health and involvement in bullying later on, is fully lacking. Children with motor problems have been recognized to be at risk for victimization [10]because impairments in motor skills seem to lead to poor psychosocial functioning and anxiety in adolescence [11-13]. Moreover, negative family factors like interparental conflict and family break up are positively associated with bullying involvement [14,15]. However, evidence on the long-term effects of family factors on bullying/victimization is limited [16].

We aimed to examine the impact of family characteristics, including parental mental health, and preschool behavioral, emotional, and motor problems on 1) being a bully 2) being a victim of bullying, or 3) being a bully/victim at ages 11 and 13.5.

## Methods

### Sample

The TRacking Adolescents' Individual Lives Survey (TRAILS) is a prospective cohort study of Dutch preadolescents from the age of 11 onwards. The TRAILS target sample involved all 11-year-old children living in the three largest cities and several rural areas in the North of the Netherlands. The present study involves data from the first (T1), which ran from March 2001 to July 2002, and the second (T2) assessment wave, which ran from September 2003 to December 2004. Of the eligible households, 76.0% (n = 2230) were enrolled in the study (i.e. both child and parent agreed to participate) at T1. Respondents and non-respondents did not differ with respect to the prevalence of teacher-rated problem behavior and the associations between sociodemographic variables and mental health indicators [17,18].

Of the 2230 baseline (T1) participants, 96.4% (n = 2149, 51.2% girls) participated in T2, 2.5 years after T1. A detailed description of the sampling procedure and methods is provided by De Winter et al [17].

### Subsample with Peer Information

Peer nominations were only assessed in classrooms with at least 10 TRAILS respondents. For this reason, only children from classrooms with at least 10 TRAILS respondents were included. Next to this, parent information on pre-school behaviour had to be available. This resulted in 982 children at T1 and 977 at T2. Mean age at T1 was 11.06 (*SD *= 0.52) and 13.5 (*SD *= 0.51) at T2. At T1 55.7% were females and at T2 52.8%. Concerning age, sex, preschool behaviors, socioeconomic strata (SES) and family breakup, the subsamples did not differ from the other TRAILS respondents; effect sizes for differences ranged from trivial to small [19].

### Measures and Procedure

*Bullying and victimization *were assessed with peer nominations at T1 and T2. Children received a list of all classmates and were asked to nominate bullies and victims among them. A definition of bullying was not provided to the children. Dutch children are familiar with the term bullying (in Dutch: 'pesten'); it is a common term and has a clearly different content than teasing (in Dutch: 'plagen'). The number of nominations children could make was unlimited. Our outcome was the aggregate of all the nominations someone received from all other classmates, i.e. both TRAILS-participants and other classmates. Peer nomination is a solid alternative to self report that has been widely used to identify bullies and victims [20]. To allow for differences in the number of respondents per class, we used the proportion of nominations. Children were classified as uninvolved, bully, victim, or bully/victim based on this. At both waves bullies were defined as scoring higher than .083 on bullying (referring to the cut-off point for the highest quartile on bullying at T1), victims as scoring higher than .067 on victimization (referring to the cut-off point for the highest quartile on victimization at T1), and bully/victims as scoring higher than the cut-off points on both [3].

*Preschool behavior *was reported retrospectively by parents at T1, using the questionnaire 'How was your child as a preschooler? (age 4-5)' [3,21]. The questionnaire contained a list of behavioral, emotional and motor items, which parents rated on a five point scale in relation to their child's peers; 1 = a lot less than average and 5 = a lot more than average. Three subscales represented preschool behaviors: aggressiveness: hot temper, disobedience, bullying, and bossiness (Cronbach's alpha, *α = 0.70, 4 items*), motor functioning: ball dexterity, ability of keeping one's balance, and making flexible movements (*α = 0.86, 3 items*) and anxiety: compulsiveness, easily depressed, anxiously, afraid to go to school, victimization, shyness, and exclusion (*α = 0.79, 7 items*).

*Family characteristics *concerned parent-reported socioeconomic status (SES) and family breakup at T1. SES was measured by income level, educational level of the father and the mother, and occupational level of both parents [22]. After standardization, the five variables were combined into one SES measure (*α = 0*.84) [3]. Family break up concerned the percentage of children that have not lived with the same parents from birth to preadolescence.

*Parental mental health *(depression, anxiety, substance abuse, and antisocial behavior) was measured by means of the Brief TRAILS Family History Interview, administered at the parent interview at T1. Each syndrome was introduced by a vignette describing its main symptoms and followed by a series of questions to assess lifetime occurrence, professional treatment, and medication use (Appendix 1). For each syndrome, parents were assigned to any of the categories 0 = (probably) not, 1 = (probably) yes, and 2 = yes, and treatment/medication (substance abuse, depression, and anxiety) or picked up by police (antisocial behavior). The scores for depression and anxiety disorder were used to construct an index for internalizing disorder. The scores for substance abuse and antisocial behavior were used to construct a familial vulnerability index for externalizing disorder. The interview has been shown to yield lifetime rates that were by and large comparable to those found by using Composite International Diagnostic Interviews (CIDI), except for fathers' rates of anxiety and substance abuse, which were relatively low [23].

### Analyses

Descriptive statistics were obtained for bullying and victimization, preschool behavior, parental mental health and family characteristics. Next, we analyzed the predictive power of early risk factors on current bullying, victimization, and its combination using multinomial logistic regression. The multinomial logistic model (MNLM) can be used to examine the effects of independent variables on a multicategory dependent variable, i.e. bullies, victims, bully/victims, and uninvolved children. With four outcomes, the MLNM is roughly equivalent to running three binary logistic regressions comparing outcomes 1 to 2, 1 to 3 and 1 to 4. In the MNLM, all of the logits are estimated simultaneously, which enforces the logical relationship among the parameters and uses the data more efficiently [24]. To interpret the outcomes of the MNLM we used marginal effects [25,26]. The marginal effect for a categorical variable is the difference between being in a given category versus all other ones. The marginal effect for a continuous variable is the effect of a variable on an outcome with one point of increase the score of the variable. The marginal effects sum up to zero per variable. We first assessed the effects of all separate variables on the outcomes. Next, we assessed the multivariate (mutually adjusted) effects of all variables that attributed univariately with statistical significance (p < 0.05).

## Results

Table 1 presents the sample regarding child characteristics, family characteristics and parental mental health at T1 and T2.

**Table 1:** Child characteristics and family characteristics

	T1	T2
	
	Mean (SD)	Mean (SD)
*Child characteristics^1^*		
Aggressiveness	2.52 (0.64)	2.55 (0.64)
Motor functioning	3.13 (0.74)	3.10 (0.74)
Anxiety	2.61 (0.62)	2.62 (0.64)
		
*Family characteristics*		
Socio-economic status family	0.07 (0.77)	0.09 (0.80)
Family breakup	20%	19%
*Parental mental health^2^*		
Externalizing problems	0.10 (0.32)	0.10 (0.33)
Internalizing problems	0.54 (0.77)	0.53 (0.78)

^1 ^range: 1-5

^2 ^range: 0-2

### Early Risk Factors of Bullying and Victimization at Age 11

Univariate analyses at age 11 revealed that bullies, victims, bully/victims, and uninvolved children differed in sex, preschool aggressiveness, preschool motor functioning, preschool anxiety, socioeconomic status (all: p < .01), and parental externalizing problems (*p *= .02). No differences were found for family breakup and parental internalizing problems.

Table 2 shows the effects of the independent variables on bullies, victims, bully/victims, and uninvolved children. At age 11, 35.5% of the 982 children was involved in bullying. Children who scored high on preschool aggressiveness were more likely to be a bully or a bully/victim and less likely to be uninvolved in bullying. In addition, children who scored high on motor functioning were more likely to be a bully, and less likely to be a victim. Children who scored high on anxiety were less likely to be a bully/victim. Boys were more likely to be a bully or a bully/victim and girls were more likely to be a victim or uninvolved. Finally, children from low SES families were more likely to be a bully, a victim, or a bully/victim and less likely to be uninvolved.

**Table 2:** Multinomial Logistic Regression on Bullying and Victimization at Age 11: Marginal Effects (and Standard Errors)

Variable	Bullies (11.9%)	Victims (15.1%)	Bully/Victims (8.4%)	Uninvolved (64.5%)
Preschool behavior				
Aggressiveness	.038 (.017)*	.002 (.019)	.051 (.014)**	-.092 (.026)**
Motor functioning	.036 (.014)**	-.051 (.017)**	.011 (.012)	.004 (.022)
Anxiety	-.018 (.019)	.038 (.021) ~	-.048 (.015)**	.028 (.028)
Being a boy	.120 (.022)**	-.091 (.023)**	.066 (.019)**	-.095 (.032)**
Socio-economic status	-.030 (.014)*	-.036 (.016)*	-.024 (.011)*	.091 (.021)**
Parental mental health				
externalizing problems	.032 (.030)	.016 (.038)	.045 (.023) ~	-.093 (.052) ~

*N *= 982; **p < .01, *p < .05, ~ *p *< .10 (two-tailed)

### Early Risk Factors of Bullying and Victimization at Age 13.5

Univariate analyses at age 13.5 revealed that bullies, victims, bully/victims, and uninvolved children differ in sex, family breakup, preschool aggressiveness, preschool motor functioning, and socioeconomic status (all: p < .01). No differences were found for preschool anxiety and parental externalizing and internalizing problems.

Table 3 shows the results on bullying and victimization at T2. Compared with T1, fewer children were involved in bullying at age 13.5 (13.3% of the 977 children). At T2 children who scored high on preschool aggressiveness were more likely to be a victim or a bully/victim and less likely to be uninvolved. Children who scored high on motor functioning were less likely to be a victim. At age 13.5, boys were more likely to be a bully or a bully/victim and girls were more likely to be uninvolved. SES at T2 had the same impact on bullies, victims, bully/victims and uninvolved as at T1. Children from intact families were more likely to be uninvolved.

**Table 3:** Multinomial Logistic Regression on Bullying and Victimization at Age 13

Variable	Bullies (5.4%)	Victims (5.8%)	Bully/Victims (2.1%)	Uninvolved (86.7%)
Preschool behavior				
Aggressiveness	.008 (.012)	.026 (.012)*	.016 (.006)**	-.050 (.017)**
Motor functioning	.013 (.010)	-.037 (.009)**	.000 (.005)	.024 (.014) ~
Being a boy	.048 (.016)**	-.001 (.014)	.029 (.010)**	-.077 (.022)**
Socio-economic status	-.018 (.009)*	-.035 (.009)**	-.017 (.005)**	.070 (.014)**
Family breakup	.012 (.021)	.034 (.024)	.022 (.015)	-.068 (.034) *

*N *= 977; **p < .01, *p < .05, ~ *p *< .10 (two-tailed)

## Discussion

The findings of this study extend current understanding of child and family predictors of later involvement in bullying. They show that early aggressiveness, good motor functioning and SES had an impact on involvement in bullying during early adolescence. Early anxiety decreased the risk of being a bully/victim at age 10/11. Children from intact families were more likely to be uninvolved at age 13/14.

### Early Child and Family Risk Factors for Being a Bully, Victim, or Bully/victim

This study confirms previously described sex differences in bullying, i.e. that boys are more likely than girls to be bullies and bully/victims [27,28]. At early adolescence, girls were more likely than boys to be victims.

Early childhood anxiety decreased the likelihood of being a bully/victim at age 10/11, but had no statistically significant effects on any of the other outcomes at ages 10/11 and 13/14. One could hypothesize that the anxiety of these children makes it very unlikely for them to become a bully at that age, even in case of victimization. The effect of this early life factor seems to diminish when the child ages, and enters secondary school.

The findings further suggest that aggressive preschoolers were more likely to be a bully at age 10/11, a bully/victim at ages 10/11 and 13/14, or a victim at age 13/14. This confirms previous cross-sectional studies that consistently showed that proactive aggression is a main characteristic of bullies [7,27,29]. Similarly, the association between early aggression and later victimization has been documented before [6,8,30]. Our findings add to the available evidence that these effects persist during the transition from primary to secondary school. Moreover, low aggressiveness seems to be very predictive for being uninvolved.

Another noteworthy result of our study is that motorically skilful preschoolers were more likely to become a bully at age 10/11 while less motorically able preschoolers were more prone to victimization at age 10/11 as well as at age 13/14. Evidence on the association of preschool motor functioning and involvement in bullying later on was limited to one study showing a positive association between physical condition and bullying [31]. Our findings indicate that these associations may have their origins in early life, likely due to the important role of motor skills in a child's emotional, behavioral and social functioning [32,33]. Motorically able children may receive more positive social feedback and recognition from peers, which is likely to improve their self-image and popularity among peers. These are frequently reported characteristics of bullies. In addition, good motor skills may provide children with physical means to bully [34].

Poor motor skills have been shown to result in poor performance in both individual and team games and sports, which may reduce children's sense of competence. This in turn reduces success within peer groups and may increase the likelihood of victimization [35]. Motorically able children may receive more positive social feedback and recognition from peers, which may improve their self-image and popularity among peers which may lead to bullying [36]. In addition, good motor skills may provide children with physical means to bully. The reverse may increase the likelihood of victimization. Third, positive features, such as motor abilities, may create a context within which negative features, such as bullying, are interpreted in a more positive light [37], again making bullying more likely. Our results also may be interpreted as that motor ability is more important at primary school (T1) than at secondary school (T2) which fits with the much higher emphasis on cognition at secondary school, compared with the stronger emphasis on play and physical activity at primary school.

Concerning family characteristics, parental SES seems to be associated with involvement in bullying. In line with previous work we found that children of lower SES were more often bullies and victims [31]. The pathways leading to this require additional study.

At both time points, parental mental health was not associated with involvement in bullying. This finding contradicts previous research into the association between certain parental mental health characteristics such as depression and the quality of peer relations of their offspring [38]. An explanation might be that the measures of bullying that we used are not affected by the mental state of the adolescent itself, which may partially reflect the parental mental state. This would imply that previous findings are due to information bias.

### Strengths and limitations of the study

Notable strengths of our study are its large population-based sample of preadolescent boys and girls, and its focus on both bullying and victimization. Next to this, its longitudinal nature makes that our findings about early risk factors are more robust.

The main limitation of the present data is that we used retrospective reports of preschool behavior. As a result, parental report may have been affected by inaccuracies in their memory. This may have introduced additional random error or recall bias, if parental memory artefacts were affected by bully/victim status. Our use of peers as informants regarding bullying makes such a bias less likely, though.

Second, parents were asked to rate their child's early behavior relative to its peers, which may be subject to recall bias. However, previous studies have shown a good parental recall of early life factors such as maternal smoking during pregnancy, maternal smoking during pregnancy, for gestational age, and for birth weight [39-41]. Despite this, our results thus need confirmation by use of prospectively collected data.

### Implications

The results of the present study imply that preschool behavioral, emotional and motor problems, and family characteristics are related to involvement in bullying at a later age. Prevention of bullying and its consequences can be enhanced by focusing on risk groups in early life.

One of the main findings of this study is the predictive value of motor performance on involvement in bullying at an advanced age. Because it is inadequate and nearly impossible to intervene on the often superior physical status of bullies, the focus has to be on children with a poor motor performance which often persist throughout adolescence and into adulthood [42]. Next to this, additional longitudinal research incorporating more detailed measures on motor performance is needed to assess the way in which motor skills affect involvement in bullying and have the potential to prevent victimization.

## Conclusions

Our results show that certain preschool behavioural problems and family characteristics are related to involvement in bullying at a later age. It shows that early aggressiveness, good motor functioning and SES had an impact on involvement in bullying during early adolescence. Early anxiety decreased the risk of being a bully/victim at age 10/11. Children from intact families were more likely to be uninvolved at age 13/14. The findings stress the importance of timely identification of at-risk children and provide the basis for targeted intervention.

## Abbreviations

TRAILS: Tracking Adolescents' Individual Lives Survey; SD: Standard Deviation; SES: Socioeconomic Status; CIDI: Composite International Diagnostic Interview; MNLM: Multinomial Logistic Model

## Competing interests

The authors declare that they have no competing interests.

## Authors' contributions

DEMCJ had the original idea for the project, wrote the initial manuscript and revised article drafts leading to the final version. RV performed the statistical analyses, which were discussed by DEMCJ and SAR. RV and SAR reviewed and edited draft manuscripts. JO and FCV supervised the data collection, and revised the final version. All authors read and approved the final manuscript.

## Appendix 1

### Vignette of anxiety complaints

Vignette: people who suffer from anxiety complaints often experience a high degree of anxiety and tension. Most people experience anxiety at some point in their lives, but in case of anxiety complaints, fear is unusually strong and intense and it is 'more-than-normal' for that person. It may concern a common sense of fear, or fear in particular situations, a phobia. It is also possible that someone suffers from anxiety panic attacks or from obsessive-compulsive ideas or actions (if necessary an clarification of the interviewer will follow).

Regarding the question on the nature of the complaints, the interviewer asks the following questions:

□ Was it a matter of continuing fear and tenseness, which had nothing to do with a particular situation? (generalized anxiety disorder)

□ Was/is he/she only anxious in social situations, afraid of looking like a fool in front of other people? (social phobia)

□ Was/is he/she anxious for other situations of things, like small spaces or specific animals? (specific phobia)

□ Did of does he/she suffer from panic disorders that occur suddenly? (panic attacks)

□ Did or does he/she suffer from obsessive-compulsive ideas or actions? That means: had he/she to perform repetitive actions, for example cleaning the house? (compulsive disorder)

## Pre-publication history

The pre-publication history for this paper can be accessed here:

http://www.biomedcentral.com/1471-2458/11/440/prepub
